# A severe case of Duchenne muscular dystrophy manifested with scoliosis and muscle contractures in 21-year-old male: a rare clinical image

**DOI:** 10.11604/pamj.2024.48.149.44563

**Published:** 2024-08-02

**Authors:** Deeplata Mendhe, Ramdinmawii Ralte

**Affiliations:** 1 Department of Community Health Nursing, Smt. Radhikabai Meghe Memorial College of Nursing, Datta Meghe Institute of Higher Education and Research, Sawangi (Meghe), Wardha, Maharashtra, India

**Keywords:** Duchenne muscular dystrophy, scoliosis, contractures, deformity

## Image in medicine

Duchenne muscular dystrophy (DMD) is a severe and progressive genetic condition that causes muscular degeneration and weakening due to a lack of dystrophin, a necessary muscle protein. Duchenne muscular dystrophy, which is typically diagnosed in early childhood, causes loss of mobility and other difficulties, having a substantial influence on the quality of life and life expectancy of people affected. It is inherited as an X-linked recessive inheritance. Here we report a case of a 21-year-old male with muscle weakness and wasting in the whole body, with severe crippling deformities and contractures with scoliosis. He had developed the symptoms of DMD at the age of 3 years and had been confined to a wheelchair for the past 11 years. He had a thin and emaciated appearance, poor nourishment with signs of malnutrition prominent bones and visible ribs, as well as reduced activity level. Deformities were seen with flexion contractures in the fingers and wrist, showing a claw-like appearance of the hand. Contractures and equines foot deformity were seen as contributing to difficulties in ambulation. There was no observable gait due to the patient´s inability to walk, and he relied on a wheelchair for mobility. He had undergone various investigations, including an enzyme test in which the creatine kinase is extremely elevated at 4800 U/L with a reference level of 24-195 U/L. Also, a genetic blood test showed the deletion of Exon 45. He is currently receiving corticosteroid tablets prednisone (1mg) and deflazacort (1.3mg) once a day and has been receiving home-based physiotherapy after completion of physiotherapy at the hospital.

**Figure 1:**
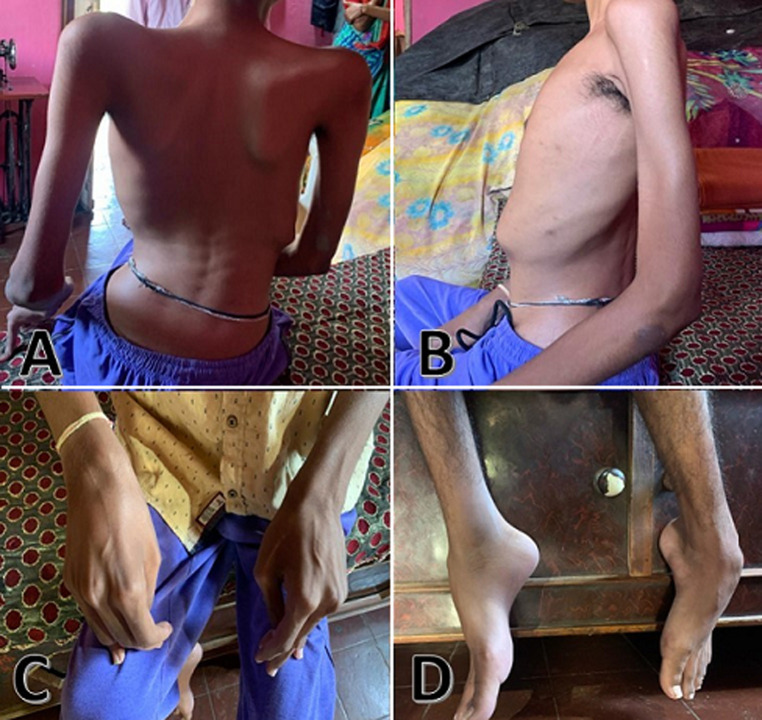
A) sideway curvature of the spine (scoliosis) with asymmetrical trunk; B) left rib cage hump on the anterior side of the chest; C) flexion contractures in the fingers and wrist, showing a claw-like appearance of the hand; D) contractures and equines foot deformity

